# Pan-immune-inflammation value predicts survival in inflammatory breast cancer patients

**DOI:** 10.3389/ebm.2025.10493

**Published:** 2025-05-01

**Authors:** Yingjia Hu, Jian Li, Mingyu Wang, Xinyi Wang, Jiankang Li, Hongfei Ji, Xingjian Niu

**Affiliations:** ^1^ Department of Medical Oncology, Harbin Medical University Cancer Hospital, Harbin Medical University, Harbin, Heilongjiang, China; ^2^ Department of General Surgery, The Second Affiliated Hospital of Harbin Medical University, Harbin Medical University, Harbin, Heilongjiang, China; ^3^ Department of Endocrinology, Health Management Center, Tianjin Union Medical Center, Nankai University, Tianjin, China; ^4^ Institute of Cancer Prevention and Treatment, Harbin Medical University, Harbin, Heilongjiang, China; ^5^ Department of Biochemistry and Molecular Biology, Heilongjiang Academy of Medical Sciences, Harbin, Heilongjiang, China

**Keywords:** inflammatory breast cancer, pan-immune-inflammation value, prognosis, biomarker, immune

## Abstract

Inflammatory breast cancer (IBC) is a rare and aggressive breast cancer subtype with poor survival. Identifying novel biomarkers is needed to predict survival for this highly progressive form of breast cancer. In this retrospective study, we investigated pan-immune-inflammation value (PIV), a novel immune-inflammation-based biomarker which combined the peripheral blood parameters (lymphocytes, monocytes, neutrophils, and platelets) in a retrospective cohort of 143 IBC patients. Then we explored the difference of PIV levels in IBC and non-IBC cohorts and the relationship between PIV and clinical characteristics in IBC patients. The survival rates of disease-free survival (DFS) and overall survival (OS) in IBC patients were analyzed and univariate and multivariate statistics were used to evaluate the prognostic value. PIV had the most significantly predictive value in IBC patients compared with other peripheral blood parameters. The mean PIV value in IBC patients was significantly higher than non-IBC patients, and the significant difference between the IBC and non-IBC was also observed in subgroups with different clinical stages and pathologic types. Furthermore, PIV performed an extensive systemic immune prognostic factor on both DFS and OS in IBC patients, and PIV was identified an independent prognostic indicator for survival outcome in IBC patients in univariate and multivariate models. Our retrospective study demonstrated the prognostic value of PIV in IBC patients, suggesting the potential application of PIV in IBC treatment outcomes. PIV would also provide some insights into the mechanisms underlying the role of immune and inflammation in IBC development and progression.

## Impact Statement

In this work, we identified pan-immune-inflammation value (PIV), a novel immune-inflammation-based biomarker, showed a significantly predictive value in IBC patients, and it's the first time to retrospectively evaluate the predictive value of PIV in IBC patients. We found that PIV had the most significantly predictive value in IBC patients compared with other peripheral blood parameters, and PIV was considered as a favorable independent prognostic indicator in IBC patients. Furthermore, the mean PIV value in IBC patients was significantly higher than non-IBC patients, which might provide some insights into the mechanisms underlying the role of immune and inflammation in IBC development and progression compared with non-IBC. Our retrospective study demonstrated the prognostic value of PIV in IBC patients, suggesting the potential application of PIV in IBC treatment outcomes.

## Introduction

Inflammatory breast cancer (IBC) is a rapidly advancing and highly aggressive form of breast cancer [1, 2]. Despite being a relatively rare subtype, IBC accounts for approximately 10% of all breast cancer related deaths [3]. Since no IBC-specific target and treatment strategy have been identified, IBC is mainly treated with the anthracycline/taxane-based chemotherapy with or without anti-HER2 therapy similar to non-IBC [4]. However, patients suffered with IBC usually have shorter survival time and worse prognosis compared with non-IBC cases [5], and there is still controversy about the prognostic evaluation of IBC. Therefore, it is essential to identify novel biomarkers to predict survival, which will contribute to make accurate treatment plans to benefit IBC patients.

Characterized by involvement of skin, the clinicopathological features of IBC are due to the lymphatic obstruction caused by widespread of tumor emboli [6]. There is evidence supporting that the contact between cancer cells and tumor microenvironment (TME) is required for the unique emboli form of IBC [6, 7]. Recent studies have revealed the contributions of the TME to the progressive and invasive behavior of IBC, such as immune evasion and chemotherapy resistance [6, 8, 9]. Among the components in TME, tumor associated macrophages are considered the main immune inflammatory cells of the TME in IBC, which usually polarize to alternatively activated M2 macrophages and act as immunosuppressive cells to induce tumor metastasis [8, 10, 11]. Besides, tumor-infiltrated lymphocytes (TILs) also participate in the controlling and transforming of TME in IBC and play a significant role in initiation and progression of tumor [7]. These individual cells within the TME collectively lead to a unique immune microenvironment of IBC, which provides a novel perspective on investigating the immune features of IBC and evaluating the IBC-associated immune inflammatory markers for forecasting the prognosis and adopting suitable therapeutic strategies.

Over past few years, a number of immune inflammatory biomarkers such as neutrophil to lymphocyte ratio (NLR), platelet to lymphocyte ratio (PLR), and monocyte to lymphocyte ratio (MLR), are based on blood parameters and easy to evaluate, which have been widely studied and showed their values in predicting the prognosis of breast cancer [12–14]. Given the complexity of the TME in IBC, the pan-immune-inflammation value (PIV) may provide additional information [14]. PIV is a kind of novel score index that combines the counts of these immune inflammatory cells involved may provide more relevant information [15–21]. It was initially used as a tool to predict survival of advanced colorectal cancer [16], and has been gradually discovered its potential value in many other cancer subtypes, especially in breast cancer [15, 17, 18, 22]. However, there is currently no available PIV data could assess the treatment efficacy of IBC. In this article, we conducted a single-center, retrospective assessment, aiming at illustrating prognostic significance of PIV in IBC patients and providing a novel immune biomarker for IBC patients.

## Materials and methods

### Patient population

Patients clinically diagnosed with IBC in the Harbin Medical University Cancer Hospital during January 2010 to December 2023 were enrolled into this current retrospective, single-center investigation. All cases (n = 143) were clinically defined according to international consensus criteria that the patient exhibited typical clinical features of IBC and fulfilled the pathological T4d diagnosis [1]. The clinic-pathological data of patients, including age, TNM stage, histopathological information (receptor subtype, pathological type, grade, Ki67 and P53 status), body mass index [BMI, as weight (kg) divided by the square of height (m^2^)] [23] and follow-up details, were collected in accordance with the ethical principles outlined in the Helsinki Declaration regarding research involving human subjects. Neoadjuvant chemotherapy was administered in stage III IBC patients, which was anthracycline-based, incorporating taxanes. Anti-HER2 targeted therapy was combined with the chemotherapy in the cases with HER2-positive status. Subsequently, patients who underwent mastectomy and axillary lymph node dissection were subjected to chemotherapy, endocrine therapy and radiotherapy. However, for stage IV IBC patients, salvage therapies including chemotherapy, anti-HER2 targeted therapy and endocrine therapy were applied according to the molecular subtypes. To evaluate whether the peripheral blood parameters were correlated with the phenotype or with advanced stage in IBC, 168 non-IBC patients were also collected between January 2010 and December 2023 at Harbin Medical University Cancer Hospital. This non-IBC group was randomly sampled to match the IBC cases in the same period and the molecular subtypes when diagnosed. Patients who underwent immunomodulatory treatment or had hematological disease and a history of malignancies were excluded. This study was approved by the Ethics Committee of Harbin Medical University Cancer Hospital, and all patients had written informed consent.

### Blood count collection

The pre-treatment peripheral blood data of neutrophil count, lymphocyte count, monocyte count and platelet count were obtained 1 week before any treatment. The absolute counts of neutrophils, lymphocytes, monocytes, and platelets were used to investigate NLR, MLR, and PLR. The PIV was calculated as neutrophil count × platelet count × monocyte count/lymphocyte count [14].

### Follow-up

Patients were regularly followed up using a system that combined telephone communication and the follow-up department. The recorded information included patients’ health status, disease progression and date of mortality. DFS (disease-free survival) was calculated as the period (in months) from disease diagnosis until disease recurrence or death. OS (overall survival) was calculated as the time (in months) from disease diagnosis to the date of death due to any cause. The last follow-up date was considered as the survival study endpoint for all patients.

### Statistical analysis

ROC curve analysis was performed to determine the cut-off values for PIV, NLR, MLR, and PLR, taking disease recurrence or death as the endpoint of interest. Correlations between high or low PIV groups and clinicopathological features were analyzed using the χ2 or the Fisher’s exact test when appropriate. Two-tailed, unpaired Student’s t tests and one way ANOVA were used to analyze the statistical significance of PIV with clinical parameters between IBC and non-IBC patients. Kaplan-Meier curves were used to visualize survival probabilities over time, the log-rank test was employed to compare survival curves between groups based on the indicator. The Cox regression analyses were conducted to evaluate the influence of clinic-pathological parameters on clinical survival outcomes. All statistical analyses were performed using SPSS 20.0 statistics software (IBM, USA). Statistical significance was defined as *p* < 0.05 (two-tailed).

## Results

### Study population

A total of 143 IBC and matched-pair 168 non-IBC patients were enrolled in our study and the clinical-pathological features were described in Table 1. The median age of the IBC patients was 54.5 years (ranging from 28 to 85 years), higher than non-IBC patients with a median age of 51.5 years (ranging from 28 to 85 years). IBC patients were classified into IIIb (69.2%), IIIc (16.1%) and IV (14.7%) stage due to the T4d diagnosis according to the TNM-UICC [24], however in non-IBC patients, 72 patients (42.9%) were in I-IIIa stage. Compared with non-IBC patients, more IBC patients had BMI ≥35 (21.7% vs. 9.5%). The percentage of the molecular subtypes, pathological type, tumor grade and P53 status were similar in IBC and non-IBC cases.

**TABLE 1:** Baseline clinico-pathological parameters of patients in IBC cohorts and non-IBC control cohorts.

Characteristics	Total (n = 311)	IBC (n = 143)	Non-IBC (n = 168)
Age, y			
Median	53.0	54.5	51.5
Range	27–85	28–85	27–82
Stage			
I-IIIa	72 (23.2%)	0 (0.0%)	72 (42.9%)
IIIb	124 (39.9%)	99 (69.2%)	25 (14.9%)
IIIc	58 (18.6%)	23 (16.1%)	35 (20.8%)
IV	75 (24.1%)	21 (14.7%)	36 (21.4%)
Receptor subtype			
HR^+^/HER2^−^	123 (39.5%)	57 (39.9%)	66 (39.3%)
HER2^+^	119 (38.3%)	55 (38.5%)	64 (38.1%)
TNBC	69 (22.2%)	31 (21.7%)	38 (22.6%)
Pathological type			
Ductal	263 (84.6%)	123 (86.0%)	140 (83.3%)
Lobular	25 (8.0%)	9 (6.3%)	16 (9.5%)
Mixed/other	23 (7.4%)	11 (7.7%)	12 (7.1%)
Grade			
1	9 (2.9%)	2 (1.4%)	7 (4.2%)
2	177 (56.9%)	80 (55.9%)	97 (57.7%)
3	125 (40.2%)	61 (42.7%)	64 (38.1%)
Ki67 status			
Median	33.0%	31.0%	34.0%
Range	0–80%	0–80%	0–80%
P53 status			
Positive	134 (43.1%)	62 (43.4%)	72 (42.9%)
Negative	177 (56.9%)	81 (56.6%)	96 (57.1%)
Body mass index (BMI)			
<25	45 (14.5%)	17 (11.9%)	28 (16.7%)
25- <30	128 (41.2%)	54 (37.8%)	74 (44.0%)
30- <35	91 (29.3%)	41 (28.7%)	50 (29.8%)
≥35	47 (15.1%)	31 (21.7%)	16 (9.5%)

P-values were shown in bold values if they had statistical significance.

### Prognostic values of blood-based biomarkers in IBC

**FIGURE 1:**
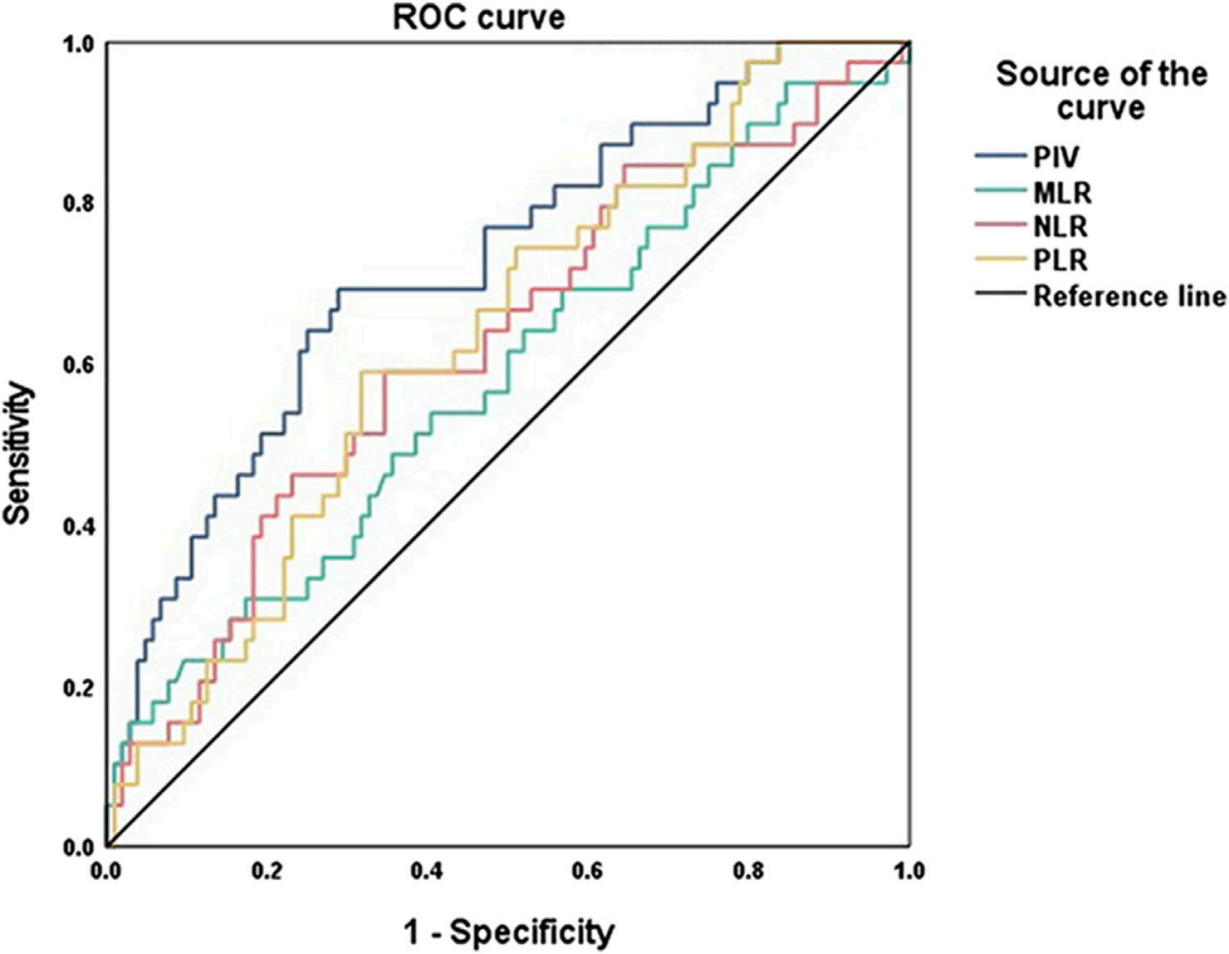
ROC curve analysis of PIV, MLR, PLR and NLR values in IBC patients.

To investigate the predictive value of immune biomarkers in IBC, ROC curve analysis was conducted and the area under curves (AUC) and cut-off values of PIV and other related biomarkers (MLR, PLR, and NLR) were shown in Figure 1; Table 2. Compared with other markers, PIV showed a better prognostic significance of IBC (AUC = 0.725, *P* < 0.001). Although PLR (AUC = 0.634, *P* = 0.014) and NLR (AUC = 0.622, *P* = 0.025) also had predictive values in IBC, the sensitivity and specificity of them were lower than PIV. Therefore, we considered PIV as the most significantly predictive value in IBC patients, and the cut-off value for the PIV was determined as 284.66.

**TABLE 2:** Predictive values of PIV, MLR, PLR and NLR on IBC survival.

Parameters	AUC	95% CI	Cut-off	Sensitivity (%)	Specificity (%)	*P-*value
PIV	0.725	0.631–0.818	284.66	69.2	71.2	**<0.001**
MLR	0.583	0.476–0.690	0.25	53.8	59.6	0.127
PLR	0.634	0.535–0.732	162.14	59.0	68.3	**0.014**
NLR	0.622	0.518–0.727	2.50	59.0	65.4	**0.025**

Abbreviations: AUC, the area under the curve; CI, confidence interval; PIV, pan-immune-inflammation-value; MLR, monocyte-to-lymphocyte ratio; PLR, platelet-to-lymphocyte ratio; NLR, neutrophil-to-lymphocyte ratio.

P-values were shown in bold values if they had statistical significance.

### PIV distinguished IBC from non-IBC cohorts

**FIGURE 2:**
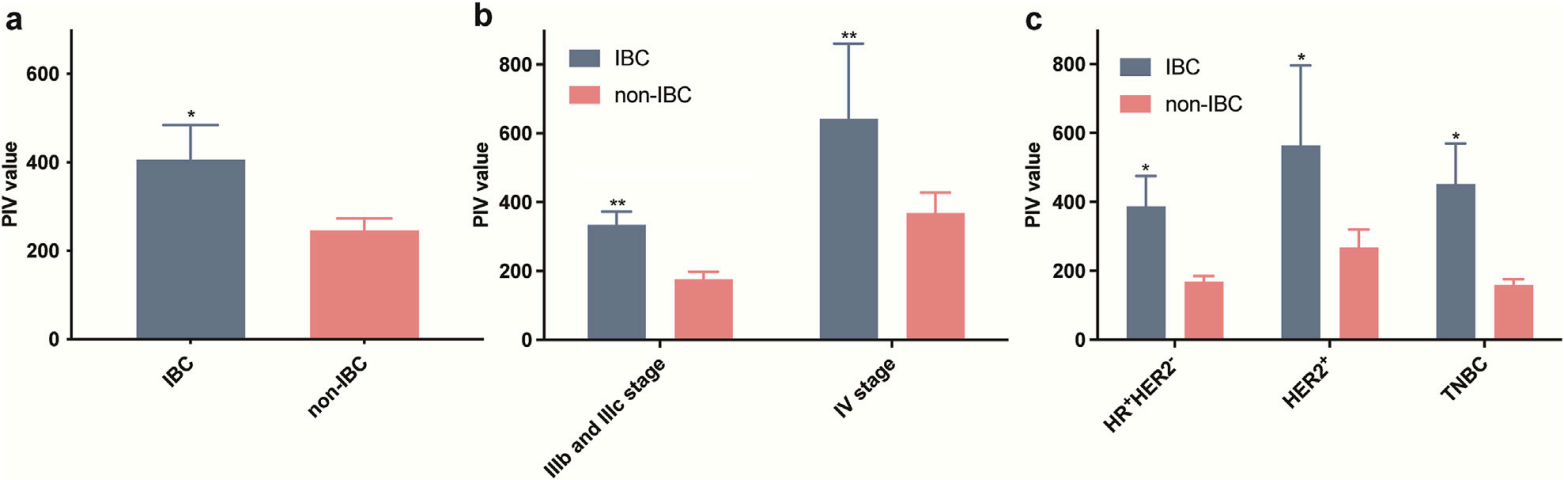
Comparison of PIV values in all IBC and non-IBC patients **(a)** as well as in different stages **(b)** and receptor subtypes **(c)** among IBC and non-IBC patients.

Since PIV was a comprehensive immune-associated biomarker, we explored whether IBC had different PIV levels compared to non-IBC cohorts. Overall, the mean PIV values of IBC were significantly higher than that in non-IBC patients (Figure 2A). Furthermore, patients were categorized by different clinical stages or receptor subtypes. The results showed that stage IV tumors had higher PIV values in both IBC and non-IBC groups, and the mean PIV values in IBC were also higher than non-IBC patients whether their tumors were stage IIIb, IIIc and IV (Figure 2B). According to receptor subtypes, the patients were classified into HR (hormone receptor)^+^HER2^-^, HER2^+^ and TNBC (triple negative breast cancer) subtypes, and we found that PIV was significantly elevated in IBC cases in all pathological types (Figure 2C). These results indicated that the PIV in IBC patients was a characteristic biomarker, which could distinguish IBC from non-IBC cohorts.

### IBC patient characteristics according to PIV value

To further investigate the role of PIV in IBC, the IBC patients were classified into high or low PIV groups according to the calculated PIV cut-off value of 284.66. A comprehensive overview of clinical-pathological features according to PIV are summarized in Table 3. In our study population, 58 (40.6%) patients presented a high PIV, which displayed a higher incidence of lobular pathological type (*P* = 0.047) and advanced tumor stage (*P* = 0.040), suggesting a more aggressive metastasis profile in high PIV group. However, there were no significant differences in age, receptor subtype, grade, ki-67 status, p53 status, and BMI.

**TABLE 3:** PIV distribution according to clinico-pathological characteristics in IBC.

Characteristics	Total (n = 143)	PIV < 284.66 (n = 85)	PIV ≥ 284.66 (n = 58)	*P*-value
Age, y				
≤50	51 (35.7%)	29 (34.1%)	22 (37.9%)	0.640
>50	92 (64.3%)	56 (65.9%)	36 (62.1%)	
Stage				
IIIb	99 (69.2%)	60 (70.6%)	39 (67.2%)	**0.040**
IIIc	23 (16.1%)	9 (10.6%)	14 (24.1%)	
IV	21 (14.7%)	16 (18.8%)	5 (8.6%)	
Receptor subtype				
HR^+^/HER2^−^	57 (39.9%)	35 (41.2%)	22 (37.9%)	0.839
HER2^+^	55 (38.5%)	31 (36.5%)	24 (41.4%)	
TNBC	31 (21.7%)	19 (22.4%)	12 (20.7%)	
Pathological type				
Ductal	123 (86.0%)	75 (88.2%)	48 (82.8%)	**0.047**
Lobular	9 (6.3%)	2 (2.4%)	7 (12.1%)	
Mixed/other	11 (7.7%)	8 (9.4%)	3 (5.2%)	
Grade				
1	2 (1.4%)	2 (2.4%)	0 (0.0%)	0.476
2	80 (55.9%)	48 (56.5%)	32 (55.2%)	
3	61 (42.7%)	35 (41.2%)	26 (44.8%)	
Ki67 status				
<20%	49 (34.3%)	30 (35.3%)	19 (32.8%)	0.754
≥20%	94 (65.7%)	55 (64.7%)	39 (67.2%)	
P53 status				
Positive	62 (43.4%)	35 (41.2%)	27 (46.6%)	0.524
Negative	81(56.6%)	50 (58.8%)	31 (53.4%)	
Body mass index (BMI)				
<25	17 (11.9%)	12 (14.1%)	5 (8.6%)	0.282
25- <30	54 (37.8%)	34 (40.0%)	20 (34.5%)	
30- <35	41 (28.7%)	25 (29.4%)	16 (27.6%)	
≥35	31 (21.7%)	14 (16.5%)	17 (29.3%)	

P-values were shown in bold values if they had statistical significance.

### Survival outcomes according to PIV value in IBC patients

**FIGURE 3:**
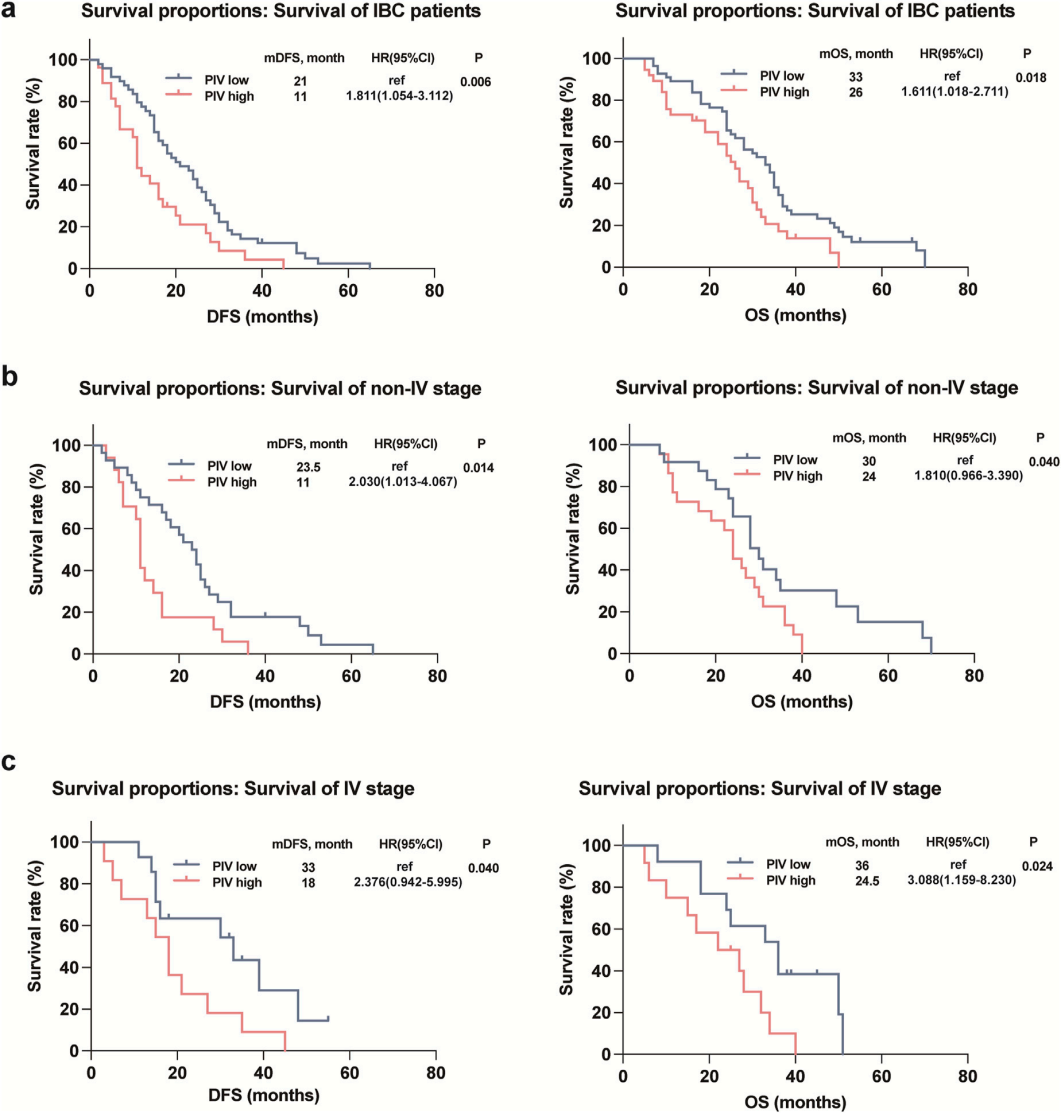
Kaplan-Meier curves for DFS and OS in the IBC patients **(a)** as well as the IBC patients with different stages **(b, c)** according to PIV.

**FIGURE 4:**
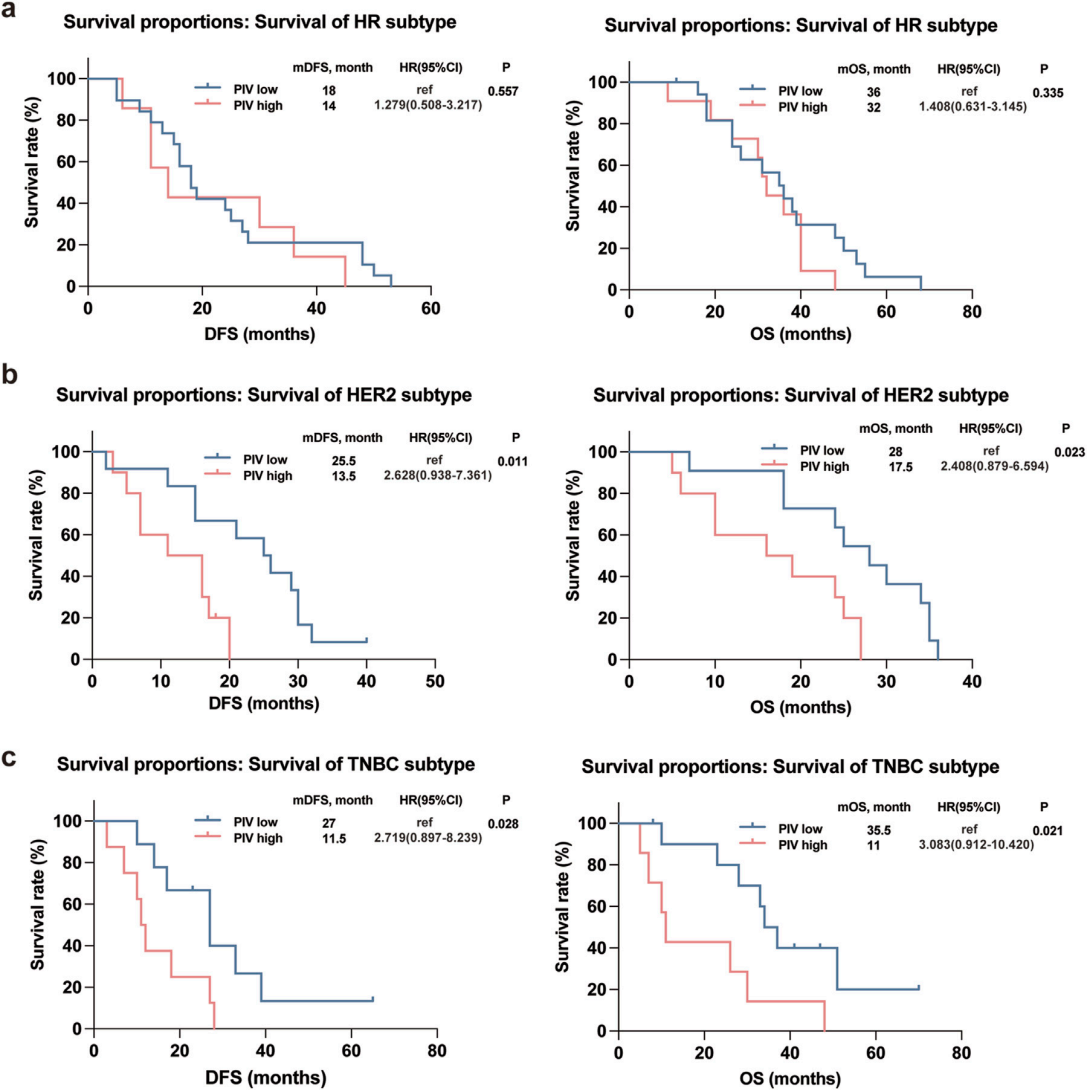
Kaplan-Meier curves for DFS and OS in the IBC patients with different subtypes according to PIV: HR subtype **(a)**; HER2 subtype **(b)**; TNBC subtype **(c)**.

Kaplan-Meier plots illustrated the relationship of PIV value with DFS and OS of IBC patients, and the results showed that the survival outcomes of IBC patients in the high PIV group were markedly worse than that in the low PIV group in both DFS and OS rates (Figure 3A). Furthermore, to better comprehend the impact of PIV on the prognosis of IBC patients in clinical stages and pathological types under various conditions, we conducted subgroup analyses and observed that similar results were observed in different clinical stages (non-IV and IV stage) of IBC patients (Figures 3B,C). However, in terms of different pathological subtypes, no significant difference in DFS or OS rates was observed in HR^+^HER2^-^ IBC patients based on high or low PIV values (Figure 4A), but HER2^+^ and TNBC IBC patients in high PIV group had statistically significantly worse survival rates (Figures 4B,C). As a result of univariate and multivariate analyses, PIV all appeared as an independent predictor in DFS and OS outcomes (Table 4). Besides, in multivariate analysis comprising the variables, MLR caused a statistically significant difference in DFS and OS survival outcomes, and P53 made a difference in DFS survival (Table 4). These results indicated that PIV was an independent value to predict survival in IBC patients.

**TABLE 4:** Univariate and Multivariate analysis of prognostic factors for survival in IBC patients.

Covariate	DFS				OS			
	Univariable		Multivariable		Univariable		Multivariable	
	HR (95%CI)	P-value	HR (95%CI)	P-value	HR (95%CI)	P-value	HR (95%CI)	*P*-value
Age, y (≤50 vs. >50)	0.971 (0.601–1.569)	0.905	0.989 (0.575–1.701)	0.969	0.972 (0.612–1.545)	0.906	1.118 (0.665–1.877)	0.674
Stage (IIIb and IIIc vs. IV)	0.883 (0.660–1.182)	0.404	0.795 (0.584–1.081)	0.143	0.931 (0.709–1.222)	0.607	0.876 (0.661–1.162)	0.359
Receptor subtype (HR^+^/HER2^-^ vs. HER2^+^ vs. TNBC)	1.052 (0.796–1.391)	0.722	1.060 (0.754–1.490)	0.736	1.115 (0.850–1.462)	0.433	1.268 (0.918–1.751)	0.149
Pathological type (ductal vs. lobular vs. mixed/other)	1.059 (0.689–1.626)	0.795	0.848 (0.503–1.432)	0.538	1.073 (0.746–1.543)	0.705	0.946 (0.620–1.444)	0.797
Grade (1 vs. 2 vs. 3)	1.062 (0.847–1.332)	0.603	1.118 (0.863–1.447)	0.398	1.054 (0.845–1.314)	0.641	1.166 (0.911–1.491)	0.222
Ki67 status (<20% vs. ≥20%)	1.139 (0.710–1.827)	0.588	1.056 (0.606–1.840)	0.848	1.057 (0.674–1.654)	0.809	0.828 (0.490–1.397)	0.479
P53 status (positive vs. negative)	1.512 (0.948–2.412)	0.082	1.965 (1.113–3.467)	**0.020**	1.142 (0.734–1.778)	0.556	1.227 (0.734–2.050)	0.435
BMI (<25 and 25- <30 vs. 30- <35 and ≥35	1.037 (0.647–1.663)	0.880	1.233 (0.698–2.177)	0.470	1.312 (0.833–2.066)	0.241	1.382 (0.837–2.281)	0.206
PIV (<284.66 vs. ≥284.66)	1.637 (1.032–2.596)	0.036	1.928 (1.098–3.387)	**0.022**	1.580 (1.017–2.455)	0.042	1.991 (1.182–3.353)	**0.010**
MLR (<0.25 vs. ≥0.25)	0.781 (0.494–1.236)	0.292	0.401 (0.211–0.762)	**0.005**	0.817 (0.525–1.273)	0.372	0.405 (0.219–0.748)	**0.004**
NLR (<162.14 vs. ≥162.14)	1.161 (0.738–1.827)	0.519	1.050 (0.504–2.187)	0.897	1.298 (0.833–2.023)	0.249	1.640 (0.885–3.037)	0.116
PLR (<2.50 vs. ≥2.50)	1.185 (0.747–1.882)	0.471	1.514 (0.772–2.967)	0.227	1.183 (0.755–1.853)	0.464	1.400 (0.770–2.547)	0.270

## Discussion

In this study, we investigated PIV, a novel immune-inflammation-based biomarker which contained the majority of immune inflammatory cell components in peripheral blood (lymphocytes, monocytes, neutrophils, and platelets), in a retrospective cohort of 143 IBC patients. First, we demonstrated that PIV had the most significantly predictive value in IBC patients. The mean PIV value in IBC patients was significantly higher compared to non-IBC patients, and the significant difference between the IBC and non-IBC was also observed in subgroups with different pathologic types and different clinical stages. Our results were in line with the well-documented perception that there was a distinct immune features and characteristic inflammation markers for IBC. Furthermore, PIV performed an extensive and favorable systemic immune prognostic factor on both DFS and OS in IBC patients, and PIV was identified an independent prognostic indicator for survival outcome in IBC patients with univariate and multivariate analyses. To our knowledge, these are novel results to estimate the prognostic value of PIV in IBC cohort.

Peripheral immune components or system is the fundamental for the orchestration and maintenance of the tumor-perturbed immune system, and also provide effective biomarkers for the diagnosis and prognosis of cancer and response to therapy [14, 25, 26]. There are studies that focusing on the immune profile of IBC, and the heterogeneous immune landscape of IBC was pointed out, which has improved our understanding of the immune characteristics of IBC [27]. In our study, PIV value in IBC patients was significantly higher compared with non-IBC patients, not only observed in different pathologic types, but also in different clinical stages, suggesting a potential distinct immune feature in IBC, that specific immune cell types of IBC may play a role in the progression and response of therapy.

Previous studies have usually used single immune component counting or the ratio of two to reflect peripheral immune system status in the prognostic modelling of IBC [12]. Lymphocytes are pivotal and multifaceted components in the anti-tumor immune response [28]. Recent research has shown that heterogeneous immune profiles in patients with IBC could impact on cancer immunity and be associated with clinical response [27]. It has been previously described that the most remarkable feature of peripheral blood in IBC was extreme lymphopenia that was highly correlated with the IBC disease itself rather than with treatment, and showed significant reduction in most subpopulations of lymphocytes [7]. As equivalently matched opponents with lymphocytes, circulating monocytes are another essential phenotype of myeloid immune cells, which are trafficked to the TME, divided into different subpopulations, and eventually contribute to local immunosuppression [29]. Previous studies have found that monocytes counting in the IBC peripheral blood was more than in non-IBC patients [11].

In contrast to the predominant contributions of the above-mentioned types, there are a couple of other components in peripheral blood, such as neutrophils and platelets [30, 31]. Neutrophils and platelets are thought to be frequently replenished from common myeloid progenitor (CMP) shared with monocytes [14]. Despite being great enrichment in peripheral immune system and accumulating in a wide range of cancer, neutrophils and platelets have been implicated to emerge as not isolated performers, but rather the key mediators and crosstalk in the cancer immune systems [30]. Previous studies demonstrated that integration index (neutrophil to lymphocyte ratio or platelets to lymphocyte ratio) was associated with tumor burden and clinical parameters, such as the optimal candidate biomarker for IBC patients [12].

Compared to previous indicators (counting or ratio) with fragmented, partial information, PIV has served as comprehensive, integrated peripheral immune biomarker incorporating the globally immune components (lymphocytes, monocytes, neutrophils, and platelets) [15–17, 21, 32]. PIV initially emerged as the prognostic indicator for patients with metastatic colorectal cancer [16], and the similar prognostic significance of PIV has been well established in various malignant tumors and immune and inflammation-related disorders [19–21]. In this study, the result showed that PIV could provide more effective value in IBC compared with other prognostic markers, MLR, NLR, and PLR. This finding was similar to the results from retrospective studies in the literature of breast cancer not only in advanced stage but also in the early-stage breast cancer patients [15, 17, 18, 22, 33]. When PIV value was applied for stratification of IBC patients, it became evident that higher PIV was associated with a more unfavorable prognosis. Our study findings indicated a pronounced inverse relationship between elevated PIV levels and both DFS and OS in patients. Moreover, based on both univariate and multivariate COX regression analyses, we observed that PIV was an independent value to predict survival in IBC patients.

The present study had some limitations. First, it was a retrospective study by single-center design, and IBC showed a relatively rare incidence, with a limited number of IBC patients, which might lead to unanticipated biases, such as selection, information and confounding biases. Second, although we excluded the patients who received immunomodulatory treatment, there existed other conditions influencing the blood-based biomarkers. Third, our study did not incorporate tumor genomic or immune microenvironmental data, which may influence IBC outcomes. Besides, although we have incorporated the metabolic indicator BMI in our study, other metabolic comorbidities such as diabetes and metabolic syndrome, which may influence systemic inflammation. Finally, more detailed analysis, such as the interplay between molecular subtype, lymphovascular invasion or host metabolic status with tumor-associated inflammation in IBC remains unexplored, which are warranting future prospective studies. Although we have employed multiple strategies to minimize the biases, further validation through randomized multicenter studies is needed to be conducted to confirm observation in this study.

## Conclusion

In summary, this translational retrospective study demonstrated the prognostic significance of PIV in IBC patients, which outperformed other blood-based immune markers, suggesting its potential application in predicting IBC treatment outcomes. Higher PIV value in IBC patients compared with subtype-matched cohort would also provide some insights into the mechanisms underlying the role of immune and inflammation in IBC development and progression.

## Data Availability

The raw data supporting the conclusions of this article will be made available by the authors, without undue reservation.
